# A Systematic Investigation of Parameters Influencing Droplet Rain in the *Listeria monocytogenes prfA* Assay - Reduction of Ambiguous Results in ddPCR

**DOI:** 10.1371/journal.pone.0168179

**Published:** 2016-12-19

**Authors:** Anna Kristina Witte, Patrick Mester, Susanne Fister, Matthias Witte, Dagmar Schoder, Peter Rossmanith

**Affiliations:** 1 Christian Doppler Laboratory for Monitoring of Microbial Contaminants, Institute of Milk Hygiene, Department for Farm Animals and Veterinary Public Health, University of Veterinary Medicine, Vienna, Austria; 2 Department of Psychology, University of Graz, Graz, Austria; 3 Institute of Milk Hygiene, Department for Farm Animals and Veterinary Public Health, University of Veterinary Medicine, Vienna, Austria; University of Hyogo, JAPAN

## Abstract

The droplet digital polymerase chain reaction (ddPCR) determines DNA amounts based upon the pattern of positive and negative droplets, according to Poisson distribution, without the use of external standards. However, division into positive and negative droplets is often not clear because a part of the droplets has intermediate fluorescence values, appearing as “*rain*” in the plot. Despite the droplet rain, absolute quantification with ddPCR is possible, as shown previously for the *prfA* assay in quantifying *Listeria monocytogenes*. Nevertheless, reducing the rain, and thus ambiguous results, promotes the accuracy and credibility of ddPCR. In this study, we extensively investigated chemical and physical parameters for optimizing the *prfA* assay for ddPCR. While differences in the concentration of all chemicals and the dye, quencher and supplier of the probe did not alter the droplet pattern, changes in the PCR cycling program, such as prolonged times and increased cycle numbers, improved the assay.

## Introduction

**Fig 1:**
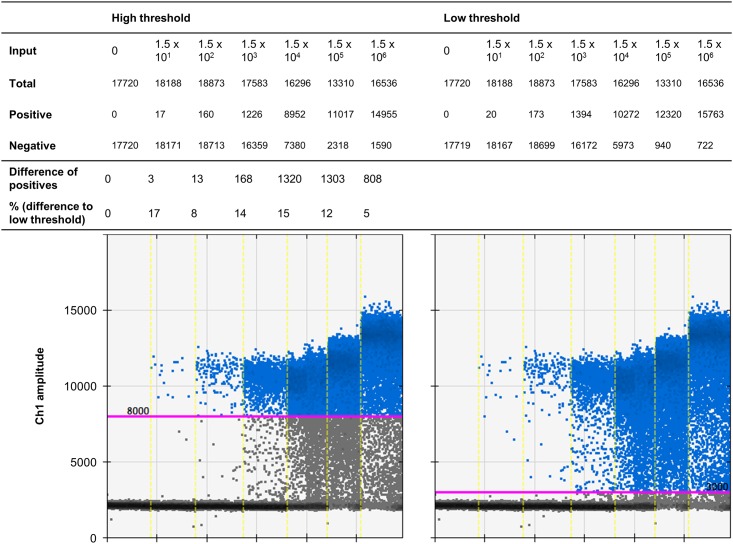
Droplets in the intermediate region. The droplet rain between positive and negative droplets prevents a clear threshold setting. Thus, the different thresholds (low or high) result in a variation of approximately 10% of the positive droplets.

Droplet digital polymerase chain reaction (ddPCR) is a relatively new method enabling quantification without external standards. It has already been established in various fields [1], such as quantification of HIV DNA [2], assessing food containing genetically modified organisms [3–6] or for the detection and quantification of bacterial pathogens in plants [7] or water samples [8]. The principle of ddPCR is based on the distribution of DNA according to Poisson distribution. In this respect, and before amplification, the reaction is divided into many small reactions (20,000 droplets in case of the Bio-Rad System) which either contain DNA or not. After the PCR run these droplets are thus either positive or negative. Knowing the number of positive/negative droplets and the Poisson distribution, the initial DNA concentration can be calculated [9,10]. Besides high precision and sensitivity, ddPCR [10] offers good inter- and intra-laboratory reproducibility [4]. Although positive and negative droplets do cluster separately, they are not always clearly divided and thus cannot be definitely classified. These droplets with intermediate fluorescence are visible in the ddPCR 1D plot as “*rain”* between the positive and negative cluster (Fig 1). The possible sources of such intermediate droplets, which have been discussed in the literature, are: (i) damaged droplets [11], (ii) coagulation of multiple droplets [12], (iii) partial PCR inhibition [13], (iv) biased amplification efficiency [7], (v) non-specific amplification [14], (vi) suboptimal PCR amplification due to sequence variances [12], or (vii) irregular droplet size [2].

Software has been developed for better evaluation of ddPCRs with droplet rain, either to improve the threshold setting, such as *ddpcRquant* [12], or software such as *definetherain* [11] that excludes intermediate droplets for analysis. If the rain is caused by biased amplification efficiency, another strategy to improve accuracy and avoid incorrect thresholds is adapting the assay to avoid or minimize the rain (and thus false positives and negatives samples). Such adaptations could be variations in the PCR program [5,6], primer concentrations [5,6], DNA digestion [15], DNA preparation [7] and others. Nevertheless, this does not necessarily mean that adaptation works for every assay and one single adjustment might not be enough.

In a previous study, we showed that quantification with the Bio-Rad ddPCR system was sufficient precise, despite suboptimal cluster formation [16]. However, samples with one single droplet with intermediate fluorescence could not be clearly defined. Thus, cluster separation must be improved in order to avoid redundant repetitions. In this study, we therefore thoroughly investigated most chemical and physical parameters to potentially reducing the rain of the *prfA* assay that specifically detects and quantifies the foodborne pathogen *Listeria monocytogenes* [17].

## Materials and Methods

### DNA isolation

DNA was isolated using the NucleoSpin tissue kit (Macherey Nagel, Düren, Germany) following protocol instructions for Gram-positive bacteria. The DNA was eluted twice with 50 μl ddH_2_O (70°C).

### DNA standard for real-time PCR quantification

1 ml of a *L*. *monocytogenes* (strain EGDe) overnight culture was used for DNA isolation. The DNA concentration was measured with the Qubit ds Broad Range Kit (Invitrogen). The copy number of the single-copy *prfA* gene was calculated using the molecular weight (1 ng of DNA equals 3.1×10^5^ copies of the genome). For the DNA digestion, 100 ng DNA were incubated with 1 μl FastDigest *Bam*HI (Fisher Scientific, Vienna, Austria) for 30 minutes at 37°C with subsequent enzyme deactivation for 5 minutes at 65°C.

### qPCR

One qPCR reaction of 25 μl final volume contained 2.5 μl of 10 x reaction buffer (Invitrogen), 3.5 mM MgCl_2_, 12.5 pmol of each primer (Table 1), 6.25 pmol of each probe (Table 1), 5 nmol each of dATP, dTTP, dGPT, and dCTP, 1.5 U of Platinum Taq (Fisher Scientific, Vienna, Austria) and 5 μl of template DNA. The *prfA* qPCR was performed as previously published in an Mx3000p real-time PCR thermocycler (Stratagene, CA, USA) with initial denaturation at 94°C for 2 minutes, amplification occurred over 45 cycles at 94°C for 15 seconds and 64°C for 1 minute [18]. All qPCRs were performed in duplicate. The data were analyzed with MxPro software.

### Primers and probes

**Table 1:** List of primers and probes used.

name	sequence	
LIP1	5`-GAT ACA GAA ACA TCG GTT GGC-3`	(Eurofins, Ebersberg, Germany)
LIP2	5`-GTG TAA TCT TGA TGC CAT CAG G-3`	(Eurofins, Ebersberg, Germany)
LIP probe2 (I)	5`-FAM-CAG GAT TAA AAG TTG ACC GCA-MGBNFQ-3`(used unless otherwise stated)	(Fisher Scientific, Vienna, Austria)
LIP probe2 (II)	5`-FAM-CAG GAT TAA AAG TTG ACC GCA-MGBEQ-3`(only used when expressly stated “other supplier”)	(Eurofins, Ebersberg, Germany)
LIP probe2 (III)	5`-FAM-CAG GAT TAA AAG TTG ACC GCA-BHQ1-3`(only used when expressly stated)	(Eurofins, Ebersberg, Germany)
LIP probe2- HEX	5`-HEX-CAG GAT TAA AAG TTG ACC GCA-MGBNFQ-3`	(Fisher Scientific, Vienna, Austria)
p-lucLm5	5`-HEX-TTC GAA ATG TCC GTT CGG TTG GC-BHQ1-3`	(Eurofins, Ebersberg, Germany)
p-lucLm6	5`-FAM-TTC GAA ATG TCC GTT CGG TTG GC-BHQ1-3`	(Eurofins, Ebersberg, Germany)

### ddPCR

**Fig 2:**
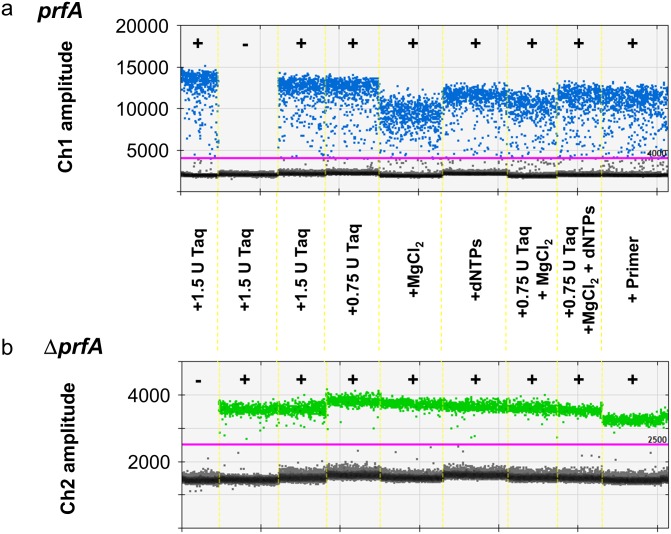
Droplet pattern with increased concentrations of polymerase, primers, dNTPs and MgCl_2_. ddPCR was performed with EGDe DNA (≈ 10^3^copies/sample, Ch1 (+)) and Δ*prfA* DNA (≈ 10^3^ copies/sample, Ch2 (+)) using modifications of the components Taq polymerase (+1.5 U, +0.75 U), MgCl_2_ (+25 nmol), dNTPs (+2.5 nmol each), Taq polymerase (+0.75 U) with MgCl_2_ (+12.5 nmol), Taq polymerase (+0.75 U) with MgCl_2_ (+12.5 nmol) and dNTPs (+2.5 nmol each) as well as increased primer concentrations (937.5 nM final concentration).

**Fig 3:**
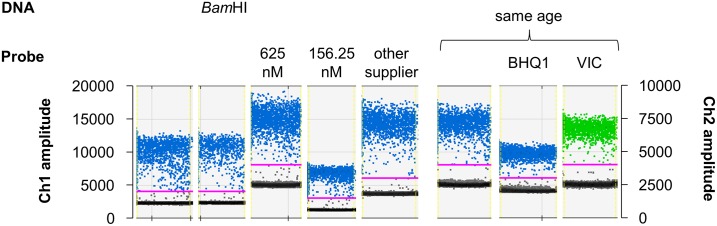
Droplet cluster with digested DNA, different probe concentrations, dyes and quenchers. ddPCR with EGDe DNA (≈ 10^3^ copies/sample) shows that the rain is similar when DNA is digested with *Bam*HI, higher or lower concentrations of the probe were applied, FAM (blue, Ch1 amplitude) or VIC (green, Ch2 amplitude) as dyes were used, MGB probes (all but “BHQ1”) or probes from another supplier were introduced. Unless indicated otherwise, 312.5 nM MGB probe with FAM as dye and the non-fluorescent quencher were used. For optimal comparison of quenchers and dyes, the last three probes were ordered and tested simultaneously.

**Fig 4:**
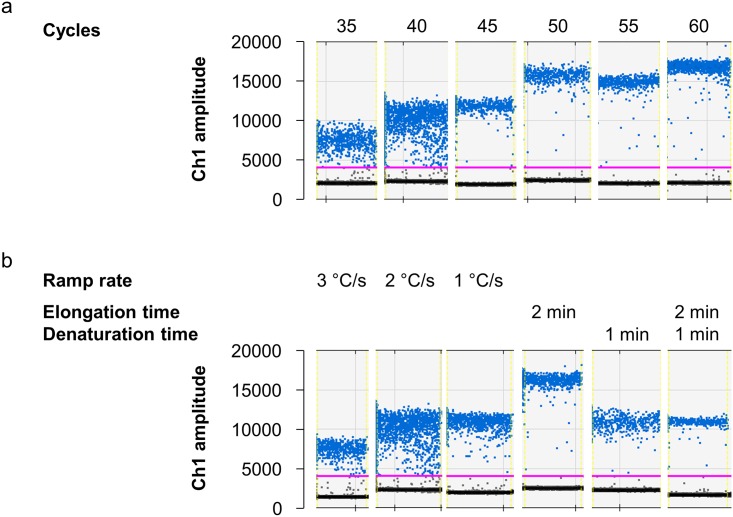
Influence of cycle number, elongation and denaturation times and ramp rate. (a) With a higher number of cycles in the PCR, separation of the positive and negative droplets is more distinct and the fluorescence level of the positive droplets higher. (b) Lower ramp rate (2°C/s or 1°C/s) promotes better droplet separation as well as longer elongation or denaturation steps do (unless indicated otherwise, one minute elongation, 30 seconds denaturation and a ramp rate of 2°C/s was used). ddPCR was performed with EGDe DNA (≈ 10^3^ copies/sample).

**Fig 5:**
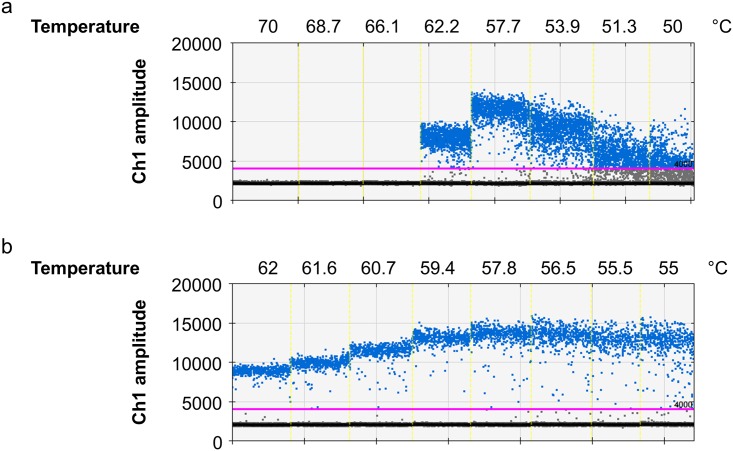
Usage of different temperatures in the PCR program. A gradient PCR between 50°C and 70°C (a) with EGDe DNA (≈ 10^3^ copies/per sample) was performed and droplets subsequently analyzed with best results at 62.2°C and 57.7°C. For more precise determination of the temperature, a gradient PCR between 62°C and 55°C (b) was performed (two minutes elongation time) with best separation of the droplets using 59.4°C.

**Fig 6:**
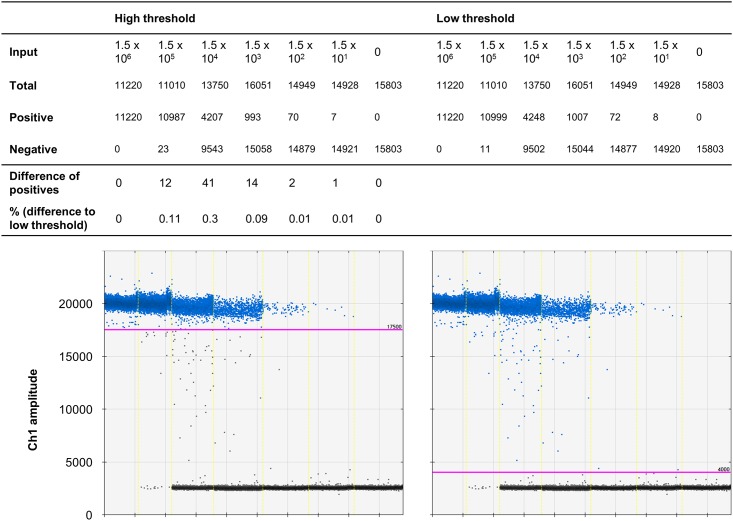
Droplets in the rain region in the optimized ddPCR. When run at 59°C, increased denaturation and elongation time (one and two minutes, respectively) and 60 cycles, the droplet rain between positive and negative droplets comprises below 1% of the positive droplets, depending on the threshold (high or low).

Unless otherwise stated, one ddPCR reaction contained 10 μl of ddPCR Master Mix for Probes (Bio-Rad, Munich, Germany), 12.5 pmol of each primer (Table 1), 6.25 pmol of each probe (Table 1), and 5 μl of template DNA. Samples were prepared in duplicate with 10% additional volume. 20 μl sample and 70 μl reader oil were transferred to respective wells in the cartridges and attached with gaskets. Droplets were generated in the QX100 droplet generator (Bio-Rad, Munich, Germany) and transferred (≈ 40 μl) to a 96-well plate and heat-sealed. Unused wells in the cartridge were filled with 10 μl of ddPCR Master Mix for Probes mixed with 10 μl H_2_O. PCR was performed as follows: initial denaturation at 95°C for 10 minutes, amplification over 40 cycles at 95°C for 30 seconds and 60°C for 1 minute and enzyme deactivation at 98°C for 10 minutes. For all steps a ramp rate of 2°C/s was used (Figs 1 and 2: T100 (Bio-Rad, Munich, Germany), Figs 3–6: C1000 touch (Bio-Rad, Munich, Germany)). Afterwards, the droplets were analyzed in the QX200 droplet reader (Bio-Rad, Munich, Germany). The data were analyzed with Quantasoft software 1.7 (Bio-Rad, Munich, Germany).

### Bacterial strains and culture conditions

*Listeria monocytogenes* EGDe (1/2a, internal number 2964) as well as Δ*prfA L*. *monocytogenes* EGDe (1/2a) were part of the collection of bacterial strains at the Institute of Milk Hygiene, Milk Technology and Food Science, University of Veterinary Medicine, Vienna, Austria. All bacterial strains were grown overnight in tryptone soya broth with 0.6% (w/v) yeast extract (TSB-Y; Oxoid, Hampshire, United Kingdom) at 37°C. Enumeration of bacterial suspensions was performed using the plate count method.

## Results and Discussion

### Occurrence of droplet rain in the *prfA* assay

As shown previously, direct transfer of a qPCR assay to ddPCR on the Bio-Rad platform is not straightforward. In the case of the *prfA* assay, we showed that direct (one-to-one) transfer resulted in strongly biased Ct values when using the chemistry (mastermix) required for ddPCR [16]. This bias was not as pronounced in the Δ*prfA* assay. The Δ*prfA* assay requires the same primers (but a different probe) and conditions as the *prfA* assay. The amplicon is shorter (100 bp instead of 274 bp) and GC contents are 49% (Δ*prfA*) and 37% (*prfA*), respectively. The two different PCR products, *prfA* and Δ*prfA*, differ only slightly in their melting temperatures (77.9°C and 78.3°C respectively).

The rain in the *prfA* assay comprises, on average, approximately 10% positive droplets leading to a quantification variation of 10%, which is acceptable for most applications (an example of one run is demonstrated in Fig 1). However, when only one or two droplets are positive, results can be interpreted as either false positive or classified as negative, because in negative controls up to three droplets are regularly found with intermediate (and high) fluorescence [2,16,19]. Thus, improved cluster separation reduces the number of ambiguous samples that must be repeated for confirmation and improved quantification.

Restriction digestion of genomic DNA guarantees separation of tandem copy genes, optimal partitioning into droplets and can reduce viscosity and thus can improve ddPCR performance (*Droplet Digital*^*™*^ *PCR Applications Guide*). *L*. *monocytogenes prfA* is a single copy gene and therefore it is not necessary to separate it by DNA digestion. Furthermore, there is no droplet rain in the Δ*prfA* assay which has the same conditions as the *prfA* assay (single copy in *L*. *monocytogenes*, same growth conditions and sample preparation). Thus, it is unlikely that DNA digestion or sample preparation are the source of the droplet rain. Yet, it has been shown by a research group who investigated two different bacteria that DNA preparation affected one ddPCR assay but not the other [7]. However, we used the same primers, worked with the same genus and chose a sample preparation method suitable for qPCR that makes it unlikely that it is the source of the rain. Nevertheless, we performed ddPCR with *Bam*HI digested DNA, which did not strongly influence the results (Fig 3). Thus, we concentrated on the chemical and physical parameters of the ddPCR.

### Conditions tested for cluster optimizations

#### Variation of chemical parameters

The *Droplet Digital*^*™*^ *PCR Applications Guide* recommends amplifying PCR products between 60 and 200 bp. Thus, the amplification of 274 bp in the *prfA* assay is rather long for ddPCR (and most qPCRs) and thus its amplification might possibly be impaired due to limitations in the PCR. One important factor for effective PCR runs is the polymerase and increased polymerase concentration can increase PCR efficiency [20]. Thus, we added polymerase that is commonly used in the *prfA* assay to improve amplification. However, supplementary addition of (0.75 U or 1.5 U) Platinum Taq polymerase (commonly used in the *prfA* assay) did not influence the droplet cluster to any extent (Fig 2). To verify that the Platinum Taq polymerase is functional in the ddPCR mastermix, we tested the Platinum Taq polymerase in the ddPCR mastermix running as qPCR (S1 Fig).

Concentrations of chemicals, such as MgCl_2_, dNTPs or primers, can also influence PCR amplification and thus could be responsible for the rain if they are limited. We routinely used the same amount of primers as in the qPCR (12.5 pmol = 625 nM). In the study of Köppel and Bucher [5], increased primer concentrations up to 800 nM resulted in much better droplet separation that was similarly demonstrated by other studies [6]. Furthermore, for ddPCR a concentration of 900 nM is recommended (Droplet Digital^™^ PCR Applications Guide). Therefore, we increased the primer concentration to 937.5 nM, but this did not improve the cluster pattern in the *prfA* assay (Fig 2).

The MgCl_2_ concentration is important for PCR, since polymerase is magnesium-dependent and primers and dNTPs bind Mg^2+^ [20,21]. Reducing the MgCl_2_ concentration in ddPCR is not possible, since the mastermix required for ddPCR comprises MgCl_2_. However, we could supplement the ddPCR with 25 nmol MgCl_2_. Nevertheless, this did not distinctly influence the droplet pattern. Interestingly, the additional MgCl_2_ even slightly reduced the maximum fluorescence values (Fig 2).

Although increasing the concentration of dNTPs has been reported to be not effective [20], we added dNTPs (2.5 nmol each) to guarantee excess of dNTPs and to exclude them as the rain source. As expected, addition of dNTPs did not improve the cluster pattern (Fig 2).

The impact of the supplemented chemicals in the Δ*prfA* assay was also rather weak. Here the addition of polymerase and primers slightly reduced the positive droplet fluorescence, the effect of the other chemicals was even lower (Fig 2), demonstrating that both assays react differently to the modifications.

Since droplets with intermediate fluorescence rarely appear in the Δ*prfA* assay, it is possible that the dye or the probe quencher, which is different in the *prfA* assay (Lip probe 2), is the source of the droplet rain. It has been shown that other assays were sensitive to probe concentrations [11]. Thus, different concentrations of the Lip probe 2 were tested, which changed the fluorescence level of both positive and negative droplets, but did not distinctly reduce the rain (Fig 3). In addition, to investigate a possible influence of the fluorophore, we replaced FAM with VIC, although FAM is commonly used for ddPCR and in other studies this channel was shown to be better [6]. As expected, the droplet rain was still present despite this exchange (Fig 3). Furthermore, minor groove binders (MGBs) coupled to the probe are frequently used in ddPCR [10]. MGBs 3’ conjugated to the probe enable the use of shorter probes with high specificity. MGBs are molecules binding in the minor groove of the DNA double strand that stabilizes the hybridization of the probe and DNA [22]. Despite the general use of MGB probes in ddPCR, we replaced the LIP probe2 MGBNFQ (MGB with non-fluorescent quencher) with a common probe with BHQ1 as quencher. This change did not improve the droplet pattern but decreased the fluorescence level of the positive droplets (Fig 3). Finally, when replacing the MGB probe with the equivalent probe of another supplier, no improvement was observed (Fig 3). All these points are arguments against the probe being the source of the rain.

However, experiments using different probes revealed a rain not as prominent as in previous experiments. The quantity of droplets in the intermediate region was still similar, but the two clusters were more distinct. For the first trials another thermocycler and droplet reader were used, indicating that physical parameters are responsible for the droplet rain. A recent study demonstrated that there is variation between thermocyclers in terms of temperatures and step length when running the same program, which influence PCR results [23]. A discrepancy was also observed between different wells that also varied between different thermocyclers of the same model. Thus, it is indispensable to investigate the thermal profile.

#### Variation of physical parameters

Besides optimization of temperatures and duration of the cycling steps, which might be impaired by using different cyclers [23], differing numbers of cycles can be tested. For our ddPCR approach, we used 40 cycles as recommended by the supplier (Droplet Digital^™^ PCR Applications Guide). Köppel and Bucher [5] showed that increased cycle numbers could result in better cluster separation. The group tested between 29 and 50 cycles and showed that more than 44 cycles did not improve the droplet pattern. In our case, an increase of cycle numbers up to 60 cycles indeed resulted in better cluster separation and higher fluorescence values of the positive droplets, whereby the difference between 50 and 60 cycles was rather small (Fig 4a). In case of the Δ*prfA* assay, the effect of the higher cycle number was clearly less pronounced (S2 Fig).

Since the Δ*prfA* assay showed good separation with 40 cycles, this result indicates biased amplification of *prfA*. Thus, we also tested different temperatures, different ramp times and longer denaturation/elongation times to improve the assay performance. Gerdes et al. [6] showed that temperature reduction can help improve droplet separation. Therefore, we tested temperatures from 70°C to 50°C using the temperature gradient block. Best separation was achieved with 62.2°C and 57.7°C (Fig 5a). Higher temperatures resulted in no positive droplets, implying no amplification at all; lower temperatures caused more intermediate droplets indicating hampered amplification. Thus, the used temperature of 60°C seems to be within the proper range for amplification. However, the rain was stronger in this gradient PCR than before. In the case of the Δ*prfA* assay, temperatures lower than 62.2°C showed a similar pattern S3 Fig.

Slower ramp rate is recommended for ddPCR and was previously shown to be important for ddPCR [16], but it cannot be changed when using the gradient program. Thus, the ramp rate is probably responsible for the stronger rain in this experiment. The pattern of using a ramp rate of 1°C/s, 2°C/s and 3°C/s is demonstrated in Fig 4b and confirms that a ramp rate of 2°C/s or slower is necessary, assuming that the amplification reaction of the ddPCR requires more time. Thus, as a next step, we doubled the combined annealing/elongation time (hereafter referred to as elongation time) and the denaturation time, independently and combined. All these changes strongly improved droplet separation (Fig 4), corroborating the theory that the ddPCR amplification reaction requires more time. Previously, Laurie et al. [24] have shown a positive influence of longer elongation times on droplet fluorescence. This group also showed that longer amplicons require longer elongation times working with fragments up to 860 bp. However, the biased amplification started only with more than 360 bp, while the 274 bp amplicon (same size as our *prfA* amplicon) was not impaired compared to the smaller fragments [24], suggesting that not only the size of *prfA* is responsible. This is in line with the observations that longer denaturation and the combination of denaturation/elongation improve the droplet pattern, suggesting that other aspects than target length are also critical.

We ultimately tested temperatures between 62°C and 55°C in the temperature gradient block (Fig 5b) with doubled elongation time (to reduce the rain and better recognize the temperature effect). Finally, the temperature associated with best droplet separation (59°C) was applied in the experiment combining all factors that reduced the rain in the *prfA* assay. The run with doubled denaturation/elongation time (one/two minutes), 2°C/s ramp rate, 59°C and 60 cycles is shown in Fig 6. Here, the rain comprises below 1% of positive droplets, showing that the combination of different adjustments is most effective. In the case of the Δ*prfA* assay, these conditions hardly influenced the droplet cluster, which is not surprising since the Δ*prfA* assay is almost optimal under standard conditions. The only obvious effect was that under these modified conditions the fluorescence amplitude is slightly higher than under standard conditions S4 Fig.

After optimization of the *prfA* assay, one PCR run takes much more time, but it greatly reduces the number of unclear samples, which minimizes repetitions. One advantage of PCR is economy of time and thus the trend is towards fast cycling protocols. However, as shown in this study and others (e.g. [25]), quality might be compromised by speed. One of probably several reasons for this is the temperature variation of different thermocyclers [23]. Since ddPCR is nevertheless much faster than microbiological methods, the time saving potential is considerable, despite the longer PCR program in the case of detecting *L*. *monocytogenes* using the *prfA* assay compared with conventional methods. Thus, the results of our study suggest that reliability and reproducibility of other ddPCR assays could also be improved by using slower cycling protocols.

## Conclusions

In summary, we extensively investigated all significant factors that could cause the droplet rain in the *prfA* assay and, as control, also in the Δ*prfA* assay. Reducing the rain improves accuracy of ddPCR and minimizes false positive and false negative results. For practical applications this means that repetitions of ambiguous results were reduced. In the *prfA* assay it was mainly cycle number and denaturation/elongation time, separately and especially in combination, together with the optimal temperature, that improved droplet cluster separation. Other factors, in contrast, hardly influenced the *prfA* assay (such as primer or probe concentrations). In conclusion, each assay must be improved accordingly, as factors not influencing the assay tested in this study might be more important for other assays as demonstrated by several researchers [5–7,11].

Thus adaption of a qPCR assay to ddPCR is laborious but necessary to improve the significance of ddPCR methodology. After multiple adjustments, the *prfA* assay has now two clearly separated clusters with only a marginal number of intermediate droplets. Since adaption of ddPCR reduces the rain to a minimum, biased amplification must be the reason for the droplets with intermediate fluorescence. Thus—at least in the case of the *prfA* assay—we can exclude non-specific amplification, damaged, coagulated or irregular droplets or sequence variances leading to suboptimal PCR amplification as possible reasons for the rain, as suggested by other researchers [2,11,12,14].

In qPCR the fluorescence signal is typically based upon the single distribution of many amplification events where any deviations from the modal amplification pattern are overwhelmed by the signal from the majority population. In contrast, the ddPCR signal is a composite of many individual amplification events where deviations may be individually manifest as rain droplets. Thus ddPCR assays show direct effects to non-optimal amplification conditions unlike related qPCR assays. Since the accuracy of both qPCR and ddPCR assays ultimately depends upon detection of signal from all targets originally present in a sample, ddPCR assays may prove to be a valuable tool for optimizing qPCR assay performance. Similar conclusions have been drawn for *chamber droplet PCR* (cdPCR), where Duewer et al. showed information that is embedded in real-time cdPCR ogive structure [26].

## Supporting Information

S1 Fig Supplemental polymerase in the ddPCR mix restores the *prfA* qPCR assay. When using the conventional PCR program the curves and Ct values are strongly delayed if the ddPCR mastermix is used (b) compared to the conventional mastermix (a). The addition of 1.5 U Platinum Taq polymerase (c) mainly restores this phenomenon. qPCR was performed with 1.5 x 10^1^to 1.5 x 10^6^copies/sample EGDe DNA (tenfold serial dilution).
(TIF)

S2 Fig Influence of cycle number, elongation and denaturation time and ramp in the Δ*prfA* assay. (a). With a higher number of cycles in the PCR, the fluorescence level of the positive droplets is slightly higher (b). Ramp rate, longer elongation or denaturation steps hardly influences droplet separation (unless indicates otherwise, one minute elongation, 30 seconds denaturation and a ramp rate of 2°C/s was used). ddPCR was performed as duplex reaction with EGDe DNA (Fig 4) and Δ*prfA* DNA (50–100 copies/sample) applied as IAC.
(TIF)

S3 Fig Usage of different temperatures in the PCR program in the Δ*prfA* assay. A gradient duplex PCR (*prfA* Fig 5) between 50°C and 70°C (a) with Δp*rfA* DNA (≈ 70 copies/sample) and between 62°C and 55°C (b, ≈ 3 x 10^3^ copies/sample, 2 minutes elongation time) was performed.
(TIF)

S4 Fig Δ*prfA* assay using standard conditions (a) and conditions optimized for the *prfA* assay (b). ddPCR of tenfold serial dilutions of Δ*prfA* DNA (1.5 x 10^6^–1.5 x 10^1^ copies/per sample) show only small differences between the standard program (a) and that optimized for ddPCR (b).
(TIF)
