# Supplementary figures and images for: A Systematic Investigation of Parameters Influencing Droplet Rain in the Listeria monocytogenes prfA Assay - Reduction of Ambiguous Results in ddPCR
Source: PLoS One. 2016 Dec 19;11(12):e0168179. doi: 10.1371/journal.pone.0168179 (PMC5167268; doi:10.1371/journal.pone.0168179)

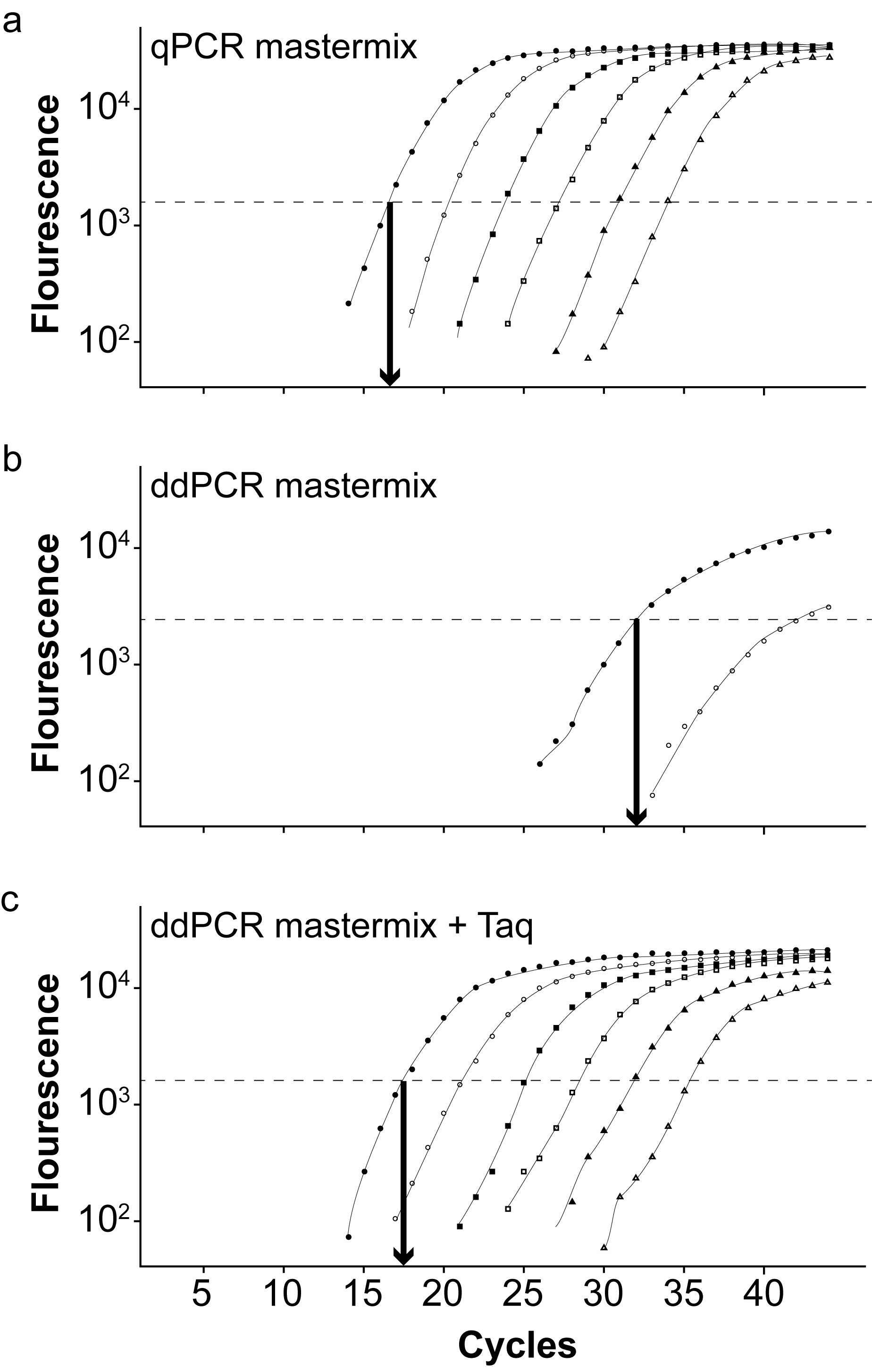

Supplement: S1 Fig — When using the conventional PCR program the curves and Ct values are strongly delayed if the ddPCR mastermix is used (b) compared to the conventional mastermix (a). The addition of 1.5 U Platinum Taq polymerase (c) mainly restores this phenomenon. qPCR was performed with 1.5 x 101to 1.5 x 10^6^copies/sample EGDe DNA (tenfold serial dilution). (TIF) [file pone.0168179.s001.tif]

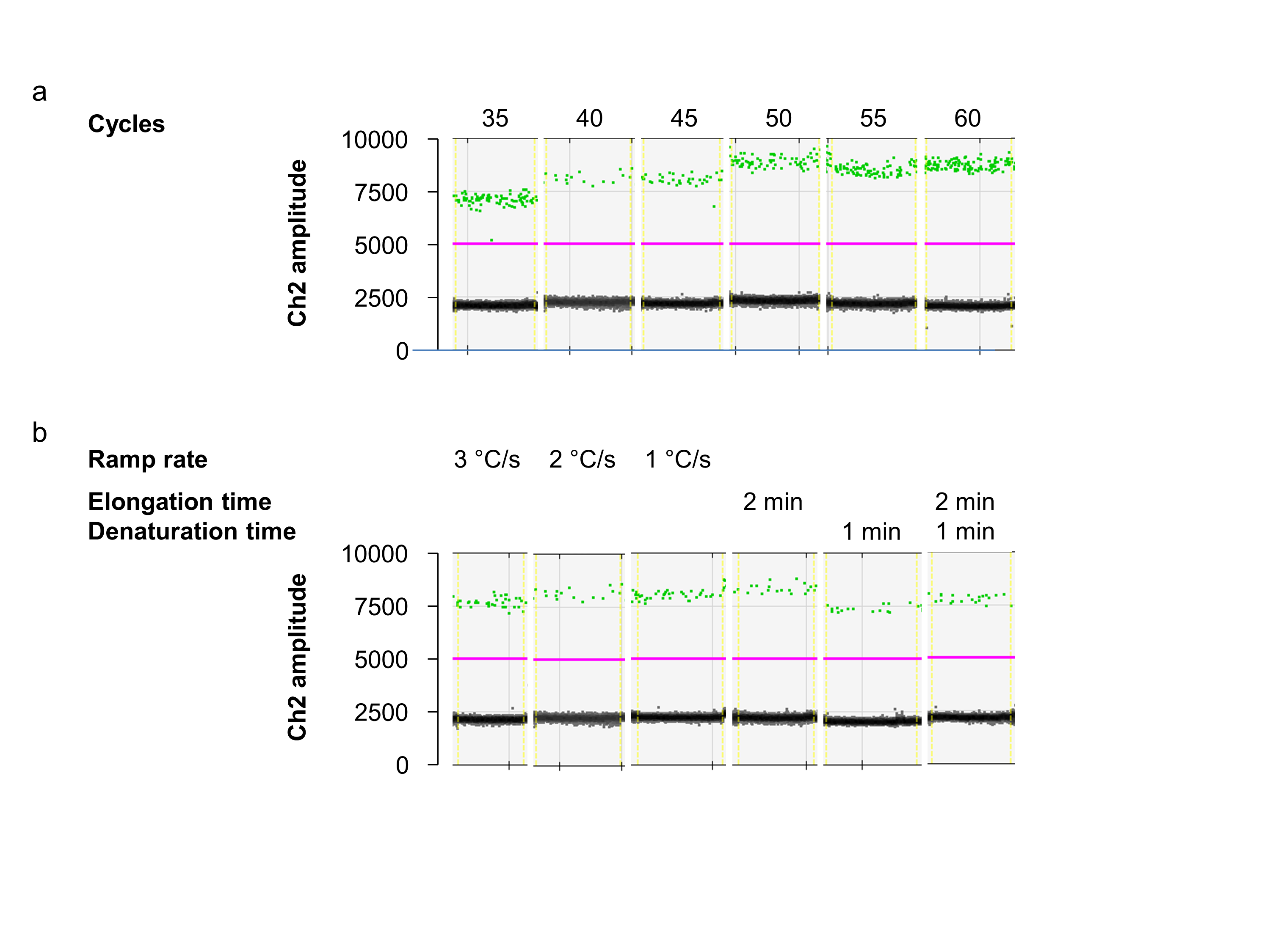

Supplement: S2 Fig — (a). With a higher number of cycles in the PCR, the fluorescence level of the positive droplets is slightly higher (b). Ramp rate, longer elongation or denaturation steps hardly influences droplet separation (unless indicates otherwise, one minute elongation, 30 seconds denaturation and a ramp rate of 2°C/s was used). ddPCR was performed as duplex reaction with EGDe DNA (Fig 4) and ΔprfA DNA (50–100 copies/sample) applied as IAC. (TIF) [file pone.0168179.s002.tif]

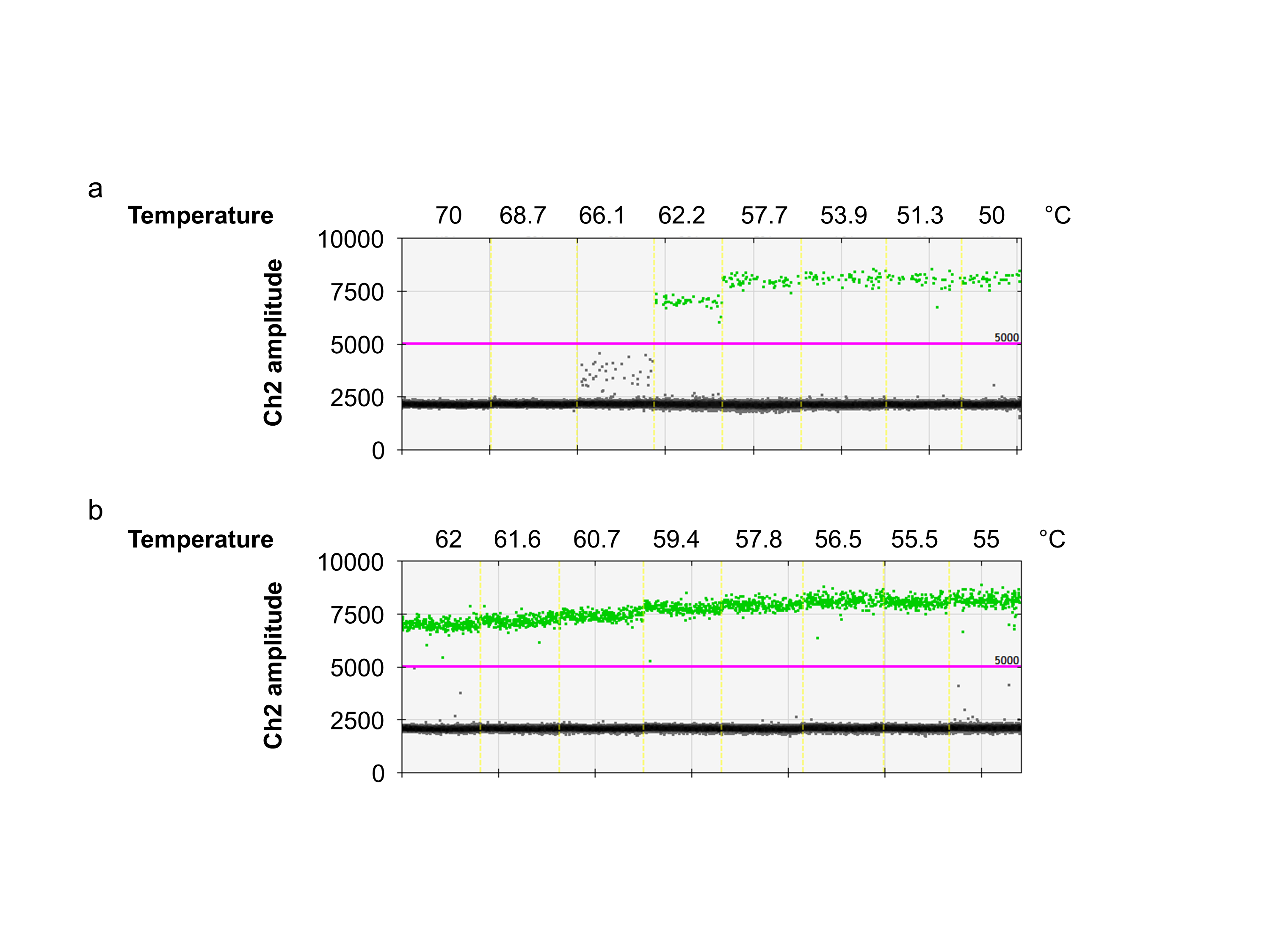

Supplement: S3 Fig — A gradient duplex PCR (prfA Fig 5) between 50°C and 70°C (a) with ΔprfA DNA (≈ 70 copies/sample) and between 62°C and 55°C (b, ≈ 3 x 10^3^ copies/sample, 2 minutes elongation time) was performed. (TIF) [file pone.0168179.s003.tif]

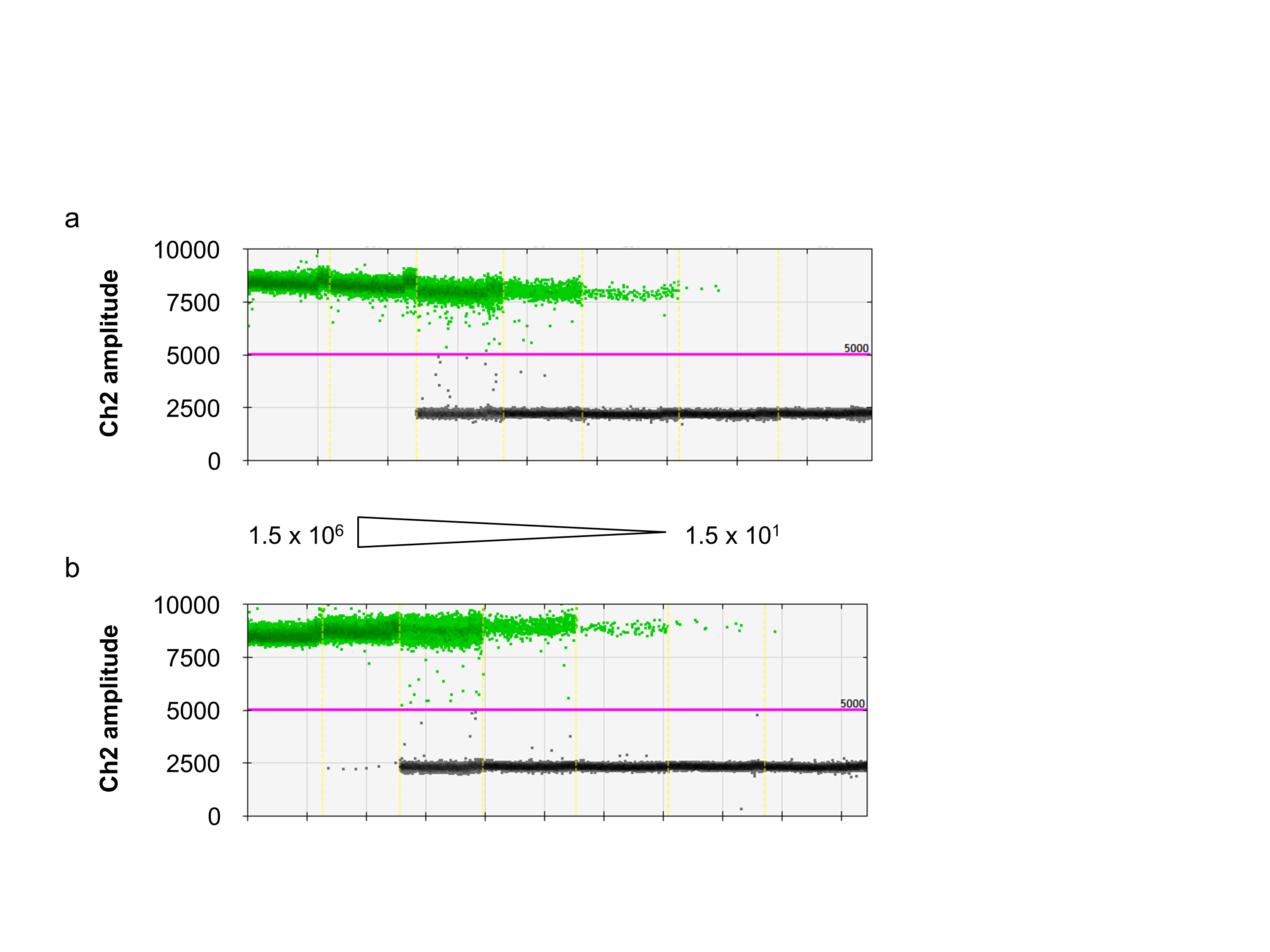

Supplement: S4 Fig — ddPCR of tenfold serial dilutions of ΔprfA DNA (1.5 x 10^6^–1.5 x 101 copies/per sample) show only small differences between the standard program (a) and that optimized for ddPCR (b). (TIF) [file pone.0168179.s004.tif]
